# Neuropsychological Findings of Extrapontine Myelinolysis without Central Pontine Myelinolysis

**DOI:** 10.1155/2007/980643

**Published:** 2007-05-30

**Authors:** Jung Im Seok, Dong Kuck Lee, Min Gu Kang, Jae Han Park

**Affiliations:** Department of neurologySchool of MedicineCatholic University of DaeguKorea

**Keywords:** Extrapontine myelinolysis, neuropsychological, central pontine myelinolysis

## Abstract

Central pontine myelinolysis (CPM) and extrapontine myelinolysis (EPM) are well recognized syndromes related to the rapid correction of hyponatremia, which are reported to show brain stem signs and various movement disorders. Cognitive dysfunction and neuropsychological findings, however, have seldom been reported. Cognitive manifestations in osmotic myelinolysis may have been underestimated due to the prominent brain stem symptoms and movement disorders. We report a case of EPM without CPM and describe the neuropsychological findings of EPM. The absence of CPM in this case made it possible to test neuropsychological function in the acute stage.

Neuropsychological testing showed severe impairment of attention, verbal and visual memory, visuospatial function, and frontal/executive function. Language and language-related functions were normal except naming.

## 1. Introduction

Central pontine myelinolysis (CPM) and extrapontine myelinolysis (EPM) are distinctive clinical syndromes with characteristic magnetic resonance features, and demyelination is frequently related to a rapid correction of an electrolyte imbalance [1,2].

Two studies have examined the cognitive aspect of CPM [3,4], and only one reported the findings of neuropsychological testing in CPM with EPM [5]. We describe a case of EPM without CPM in which neuropsychological testing showed cognitive impairment in multiple domains.

## 2. Case report

A 69-year-old man was admitted to the hospital with complaints of dizziness over a 1-week period. The sensation of imbalance was aggravated by walking, but nausea or vomiting did not accompany the dizziness. Three days prior to admission, the patient lost his balance and fell down. He did not lose consciousness. His medical history was unremarkable except for a 15-year history of hypertension that was currently being treated with diuretics (started four weeks earlier). He smoked 20 cigarettes a day for 30 years and had no past history of chronic alcohol abuse. Prior to admission, he was a manager of his own company and had 16 years of education.

On examination, he was alert and well oriented, and an ataxic gait comprised the only abnormal finding. Laboratory evaluation revealed severe hyponatremia (100 mEq/L) and a low potassium concentration (3.0 mEq/L). Other routine biochemical test results, including a white blood cell count with differential, hemoglobin, liver enzyme levels, renal function, and glucose, were all within normal limits. An ECG showed no abnormalities. We diagnosed diuretic-induced hyponatremia, and the patient's hyponatremia was corrected to 126 mEq/L over 2 days.

ISSN 0953-4180/07/$17.00 © 2007 – IOS Press and the authors. All rights reserved

Table 1
Results of neuropsychological tests in the patient

| Cognitive domain | Results | Cognitive domain | Results |
|---|---|---|---|
| Attension | | Memory | |
| digit span: forward | 3 (<1%ile) | Rey CFT: immediate recall | 3 (<1%ile) |
| backward | 2 (1.5%ile) | 20-min delayed recall | 0 (<1%ile) |
| Language and related functions | | recognition | $7 - 9 = -2$ |
| fluency | NL | Frontal/Executive function | |
| auditory comprehension | NL | motor impersistance | NL |
| repetition | NL | contrasting program | NL |
| naming (K-BNT) | 34/60 (<1%ile) | go-no-go test | NL |
| reading | NL | fist-edge-palm | NL |
| writing | NL | alternating hand movement | NL |
| calculation | NL | alternating square and triangle | Deformed |
| finger naming | AB | Luria loop | Deformed |
| right-left orientation | NL | semantic word fluency: animal; supermarket items | 2;0 (AB) |
| body part identification | NL | phonemic word fluency: sum of three consonants | 0 (AB) |
| limb praxis | NL | K-CWST: word reading: correct/incorrect | 33/4 (AB) |
| Visuospatial functions | | color reading: correct/incorrect | 30/8 (AB) |
| K-MMSE: interlocking pentagon | NL | General cognitive index | |
| Rey-Osterrieth Complex Figure Test (Rey CFT) | 5.5/36 (<1%ile) | MMSE | 19/30 |
| Memory | | CDR | 1 |
| K-MMSE: registration | 3 | GDS | 4 |
| recall | 2 | Neuropsychiatric symptoms | |
| HVLT: free recall (1st; 2nd; 3rd) | 4/12;4/12;4/12 (5.5%ile) | Geriatirc Depression Scale | 12/30 |
| 20-min delayed recall | 0 (<1%ile) | K-NPI | 2/144 |
| recognition (true positive-false positive) | $10 - 7 = 3$ | | |

K-BNT: The Korena version of the Boston Naming Test, HVLT: Hopkins Verbal Learning Test (Korean version),
K-CWST: Korean Color Word Stroop Test, K-MMSE: Mini-Mental State Examination(Korean version),
CDR:Clinical Dementia Rating Scale, GDS:Global Deterioration Scale, K-NPI: Neuropsychiatric Inventory (Korean version),
NL: within normal limit, AB: abnormal.

On the seventh day after correcting the sodium level, the patient developed progressive dysarthria, dysphagia, and a gait disturbance. On examination, he was alert, but would stare at the speaker's eyes with a fixed gaze and reduced blinking. Muscle strength was symmetrical and nearly normal in all four extremities, and reflexes were normal in all four extremities. No pathologic plantar responses were observed. Lead pipe rigidity was present, and the patient could not sit or stand without support.

We performed a brain MRI to confirm osmotic myelinolysis because parkinsonian features had occurred after the correction of hyponatremia. A T2-weighted and fluid-attenuated inversion recovery brain MRI showed symmetric high signal intensities in the bilateral caudate nucleus and putamen (Fig. 1A, B). No abnormal signal intensity occurred in the pons (Fig. 1D-F). The diffusion-weighted imaging (DWI) showed high signal intensities in the corresponding areas (Fig. 1C). The Apparent Diffusion Coefficient map showed no definite abnormality. A clinical and radiological diagnosis of EPM was made.

Since steroid administration or symptomatic treatment with dopaminergic compounds has proved beneficial in several cases [6,7], we started steroid pulse therapy that was continued for 3 days and was followed by medication with a dopaminergic agent. After the initiation of treatment, the patient's symptoms slowly improved. On the fifth day of treatment, the patient's muscle strength was normal; the dysarthria was also much improved. However, the rigidity persisted, and reflexes were increased in all four extremities. He could stand with support and could walk with substantial difficulty due to a short stride and postural instability.

Neuropsychological examination was performed on the tenth day of treatment. The results of neuropsychological testing are presented in Table 1. In summary, the patient was severely impaired in regard to attention, naming, verbal and visual memory, visuospatial function, and frontal/executive function. The patient demonstrated impaired performance on tests of recognition memory as well as free recall memory. Language and language-related functions were normal except naming. No prominent psychiatric symptoms were observed.

## 3. Discussion

Osmotic myelinolysis may develop after a rapid correction of hyponatremia. Due to its vulnerability to

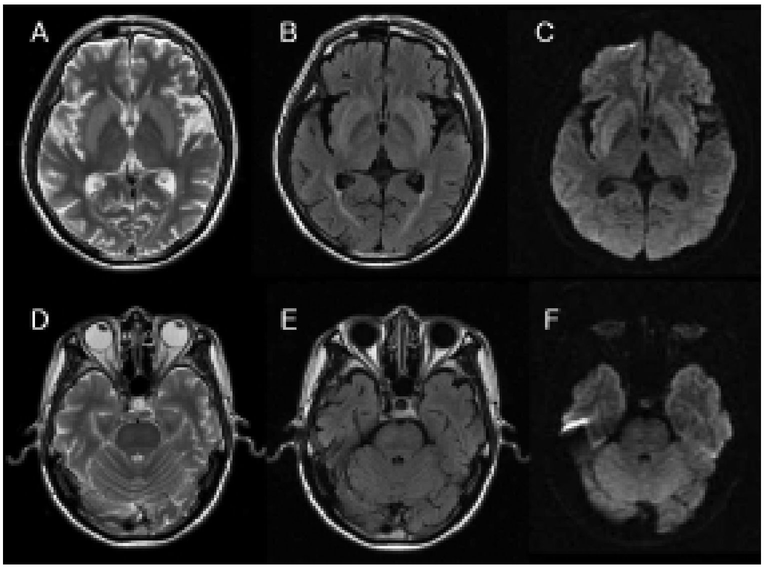

Fig. 1. Brain magnetic resonance imaging (MRI) scans. T2-weighted images show abnormal high signal intensities in the caudate nucleus and putamen (A). Fluid-attenuated inversion recovery and diffusion-weighted images show high signal intensities in the corresponding areas (B and C). There are no abnormal signal intensities in the pons (D–F).

a rapid change in electrolyte balance, the pons is the most frequently affected region of the CNS. However, myelinolysis may affect other CNS regions such as the basal ganglia and thalamus. Rarely, extrapontine, basal ganglia myelinolysis may occur in the absence of central pontine myelinolysis [8].

With careful correction of hyponatremia, osmotic myelinolysis might be prevented, though not in every case. Correction of symptomatic hyponatremia should not exceed a rate of 1–2 mmol/L/h and never more than 8 mmol/L per day [9]. In our patient, the hyponatremia was corrected more rapidly than is generally advised.

Our patient's symptoms developed after a rapid correction of hyponatremia and can be explained by the lesions found on the MRI. Although similar lesions on a brain MRI can be caused by Wilson's disease, CO poisoning, Leigh's syndrome, and Creutzfeldt-Jakob disease [10–13], the clinical history and course strongly suggested EPM. In this case, although the lesions found on the MRI appears to be predominantly involve the basal ganglia, it is possible that mild dysfunction of white matter elsewhere has occurred. More sensitive techniques such as diffusion tensor imaging may have revealed dysfunction elsewhere.

Abnormalities of the basal ganglia contribute to a variety of neuropsychological dysfunction. Frontal dysfunction and memory impairment in patients with a striatal lesion have been studied extensively in those with progressive supranuclear palsy, Huntington's disease, and Parkinson's disease [14–16]. The role of the basal ganglia in visual object and visuospatial cognition has been demonstrated [17]. Because basal ganglia is most frequently affected region in EPM, common frontal-striatal dysfunction is expected. However, studies on EPM are mostly confined to abnormal movement disorders [18,19]. Two reports have described cognitive and emotional dysfunction in CPM [3,4] and suggested that the brain stem plays a role in higher cognitive processes. Only one report has described the findings of neuropsychological testing in CPM with EPM [5]. In severe CPM or CPM with EPM, tests on cognitive function are impossible due to the prominent brain stem symptoms of paraplegia, ataxia, and reduced consciousness. The absence of CPM in this case made it possible to test neuropsychological function in the acute stage.

The present case showed neuropsychological manifestations of a basal ganglia lesion in osmotic myelinolysis. The cognitive manifestations of this disease have been underestimated due to brain stem symptoms and movement disorders. We suggest that neuropsychological deficits are an important manifestation of osmotic myelinolysis, especially EPM, and careful assessment is needed to disclose a cognitive dysfunction associated with this clinical syndrome.
